# Effects of Cold Irrigation on Early Results after Total Knee Arthroplasty: A Randomized, Double-Blind, Controlled Study

**DOI:** 10.1097/MD.0000000000003563

**Published:** 2016-06-17

**Authors:** Zhirui Li, Daohong Liu, Jiyuan Dong, Long Gong, Yong Wang, Peifu Tang, Yan Zhang

**Affiliations:** From the Department of Orthopedics, Chinese PLA General Hospital (ZL, JD, PT); Department of Orthopedics, the 309th Hospital of PLA, Beijing (DL); Department of Orthopedics, the 252th Hospital of PLA, Baoding (LG); Department of Special Medical Treatment, Beijing Shijitan Hospital Affiliated to Capital Medical Hospital (YW); and Department of Rheumatology, Beijing Jishuitan Hospital, Beijing (YZ), China.

## Abstract

Several studies have indicated that pain peaks at 24 to 48 hours after total knee arthroplasty (TKA) surgery. TKA has been associated with disruption in normal sleep patterns, swelling knee, and significant blood loss. However, a satisfactory regime to resolve these mentioned problems has yet to be found.

In this study, a total of 420 patients were randomly allocated into two groups and treated with continuous irrigation of either 4000 mL cold saline with 0.5% epinephrine or normal temperature solution. Clinical outcomes including pain scores at rest during postoperative three days, drainage output, analgesic consumption, decreased hemoglobin, sleep quality, and satisfaction rate were analyzed. Mean scores and postoperative change in scores were calculated.

Visual analog scale (VAS) pain scores in the treatment group were significantly reduced from 4 hours (*P* = 0.0016) to 24 hours (*P* = 0.0004) after TKA. Additional benefits including reduced analgesic consumption, improved satisfaction rate, and sleep quality were observed. In addition, a significant reduction in blood loss reflected by decreased Hb and drainage was found.

In this study, irrigation with a cold 0.5% epinephrine solution was a beneficial and cost-effective treatment that decreased acute postoperative VAS pain scores immediately after and 1 day after surgery. Patients reported postoperative improvement in sleep quality and overall satisfaction rate with a decrease in morphine usage. In addition, a reduction of intraoperative blood loss might decrease the blood transfusion rate and related costs. Collectively, irrigation with cold 0.5% epinephrine offers a safe, simple, and effective treatment that might improve recovery and enhance quality of life of patients undergoing TKA.

## INTRODUCTION

Total knee arthroplasty (TKA) is a well-established procedure for end-state arthritis of the knee that has been demonstrated to improve postoperative recovery and pain relief. Most patients reported improvements in pain, function, and health-related quality of life (QOL) within 3 to 6 months after surgery.^1^ Despite these improvements, patients treated with TKA often experience swelling, and pain that often peaks 24 to 48 hours after surgery, has yet to be found.^2^ In addition, many patients report disruption in quality of sleep, which often is associated with postoperative swelling and significant blood loss.^3–5^ Postoperative pain and swelling can be explained by elevated general and local inflammatory mediators.^6,7^ Thus, a reduction in the inflammation reaction might improve perioperative pain management following TKA.

Irrigation is necessary during the resection process of TKA to remove bone and soft tissue debris, potential bacteria colony, blood, and heat.^8^ The clinical benefits from the use of additives in irrigation solutions, such as antibiotic, detergent, and hypertonic saline, has been previously described.^9–11^ Continuous irrigation with certain additives have been shown to reduce bacterial growth in periprosthetic joint infection (PJI).^12^ Therefore, irrigating the wound during orthopedic surgical procedures, such as TKA, might improve surgical outcomes and reduce perioperative complications at a low cost.

Previous studies of postoperative cryotherapy have found a reduction in inflammatory cytokines that changes tissue metabolism by decreasing enzymatic function, inhibiting the stretch reflex, and reducing muscle spasm.^13^ In addition, cold stimulation could reduce bleeding that is caused by capillary oozing, medullary cavity, and bone bed interface. However, the direct benefits of postoperative cryotherapy are unclear because of the lack of standardization in instruments, frequency, duration, and temperature.^2^ Few studies have evaluated intraoperative cryotherapy in TKA. Therefore, in this study, we assessed the clinical outcomes in patients undergoing TKA who had received intraoperative chilled irrigation solution containing epinephrine to reduce the perioperative inflammation response. We hypothesized that patients in the treatment group would have less swelling and blood loss, reduced postoperative pain, and an overall higher QOL during hospital admission.

## MATERIALS AND METHODS

### Study Design

A randomized, double-blind, controlled, and single-center study was performed between May 2012 and May 2014 in the orthopedics department of the Chinese PLA General Hospital. Participants undergoing primary, unilateral TKA were placed randomly into either the treatment or control group using computer-generated randomization sequence (SAS Statistical Software 9.1.3). Before enrollment, all of the patients gave informed consent and this study was approval by Institutional Review Board and ISRCTN registry (How to recover from TKA with less pain NO.ISRTCN14054997). Sample size estimation was based on detecting the difference in visual analog scale (VAS) pain scores. The estimated number of >150 patients in each group was enough to detect a 20% difference between groups with alpha set at 0.05 and beta 0.1. An additional 10% of total participants were planned in each group to compensate for any unforeseeable loss.

### Subjects and Intervention

In this study, we enrolled a total of 420 patients undergoing primary, unilateral TKA because of degenerative knee joint osteoarthritis. The exclusion criteria in this study were the following: (1) Refuse to participate in this trail, (2) history of severe hypertension or cardiovascular diseases, and (3) Allergies to epinephrine and cold intolerance. During the procedure, all of the patients had intraoperative continuous irrigation. Patients in the treatment group received 4000 mL 4°C cold solution with 0.5% epinephrine and those in the control group were irrigated with 4000 mL of normal saline at normal temperature. Previous studies have recommended the 4000 mL solution as an effective volume for pulse irrigation to remove particles and other debris during cemented TKA. Before surgery, the treatment solution remained 4°C in a nearby refrigerator and was transported by circulating nurse at the appropriate time. Epinephrine was added to the cold solution following preparation of the bone bed. Normal irrigation saline was stored at 21°C to 24°C. After closing the joint capsule, 50 mL of normal saline or cold irrigation solution with 0.5% epinephrine was administered through a drainage tube placed in the knee joint cavity to prolong the effect of the cryotherapy treatment. The drainage tube remained clamped for 4 hours after injection. Acute postoperative pain management for all of the patients included a 48-hour patient-controlled analgesic (PCA) pump and diclofenac sodium (50 mg, tid) and parecoxib (40 mg, qd). Following hospital discharge, tramadol was given based on patients’ demand. The thromboprophylaxis protocol was 10 mg rivaroxaban every day.

### Study Parameters

The primary efficacy parameter was based on changes in the pain score using a 100 mm VAS, which ranged from 0 (standing with no pain) to 100 (worst imaginable pain). The VAS score was measured at 4, 8, 12, 16, 20, 24, 36, 48 and 60 hours, respectively, while the patient rested in bed. Patients reported postoperative sleep quality and satisfaction rate using the VAS. Local swelling and inflammation was evaluated by measuring the knee joint girth at 1 cm proximal to the upper border of the patella before surgery, and 48 and 96 hours following the procedure. The Each measurement was performed twice and the mean value was reported. Drainage output was recorded and compared following extraction of the drainage tube after 24 hours. Drainage consisted of mainly blood. The 50 mL of irrigation solution infused perioperatively was subtracted from the total volume of drainage output. Analgesic consumption depended on total doses of morphine used by PCA treatment, weak opioid analgesic, and nonsteroidal antiinflammatory drugs (NSAID) in every 12 hours.

### Surgery

The TKA procedure was completed under epidural anesthesia or nerve block by two experienced surgeons. All of the patients were given prophylactic antibiotics (vancomycin or cefotaxime) before the procedure. Cruciate ligament-retaining prosthesis (Gemini. Link, Germany) were provided for all participants in this study. The drainage tube was extracted at 24 hours following surgery. A tourniquet was applied for 30 minutes at 300 mg Hg before the femur osteotomy and after the tibia osteotomy, and released following the closure of the joint capsule.

### Statistical Methods

The significance threshold was defined using a *P*-value of 0.05. Continuous variables were shown as mean and standard deviation (SD) values. Between-group comparisons of VAS pain scores, knee girth, analgesic consumption, sleep quality, satisfaction rate, and drainage output were completed using the Student *t* test. Qualitative data was compared using a chi-square test. All values were assumed to have normal distribution. Statistics analysis was performed by SAS Statistical Software 9.1.3.

## RESULTS

**FIGURE 1:**
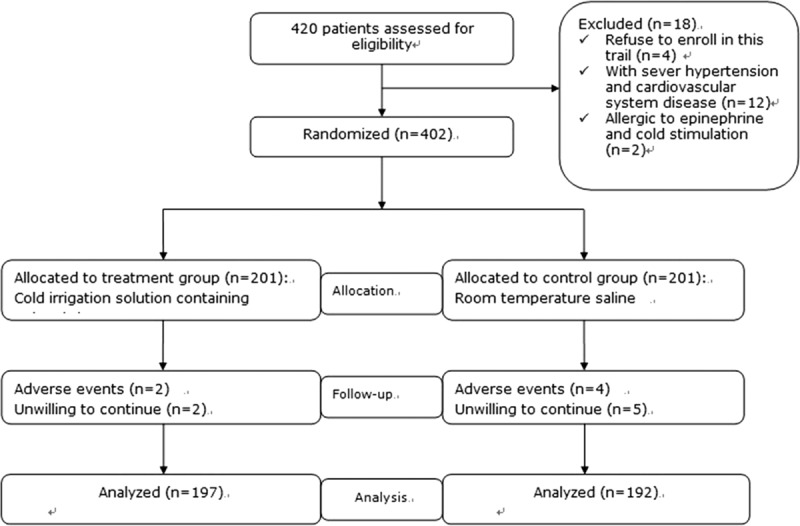
Flowchart of enrolled patients.

The initial 402 participants were randomly allocated in this study according to the exclusion criterion. After 13 patients dropped from the study (treatment group n = 4; control group n = 9) a total of 389 patients were analyzed at the end of the study (Figure 1). Baseline information and demographic characteristics are detailed in Table 1.

**TABLE 1:**
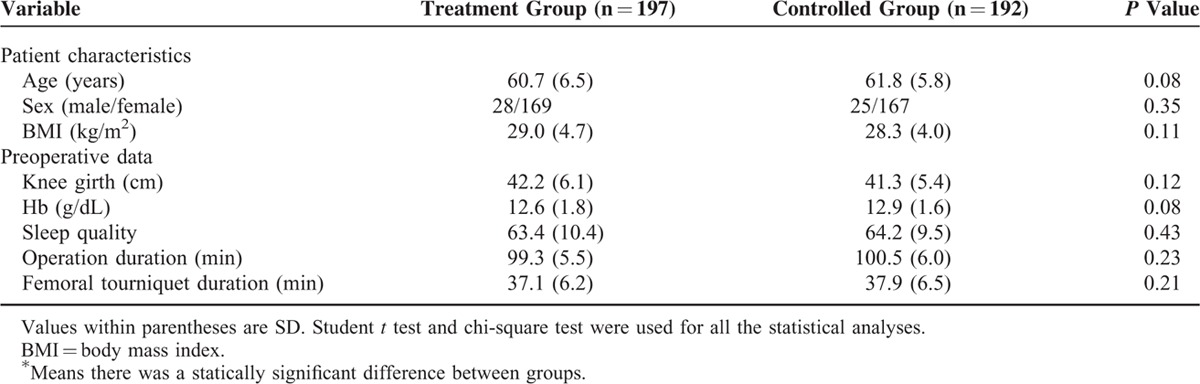
Demographic Characteristics and Baseline Information

### VAS Pain Scores and Analgesic Consumption

**FIGURE 2:**
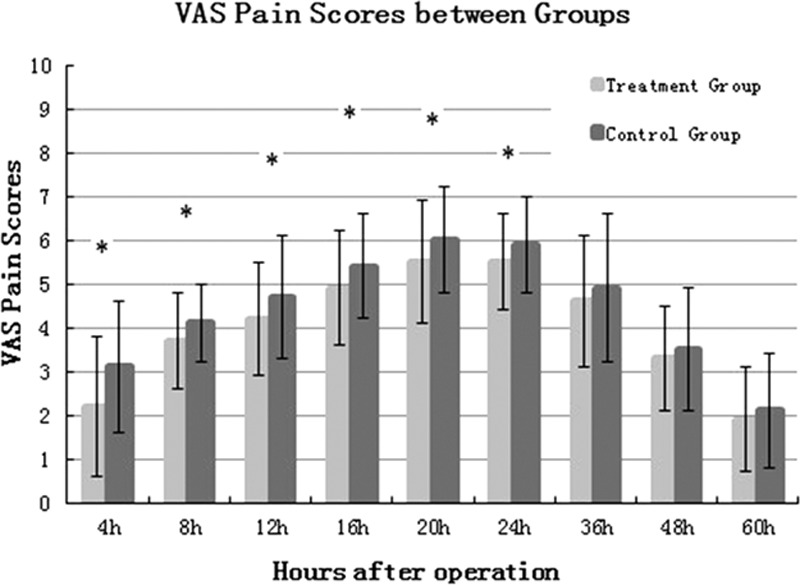
VAS pain scores between two groups. VAS = visual analog scale.

**FIGURE 3:**
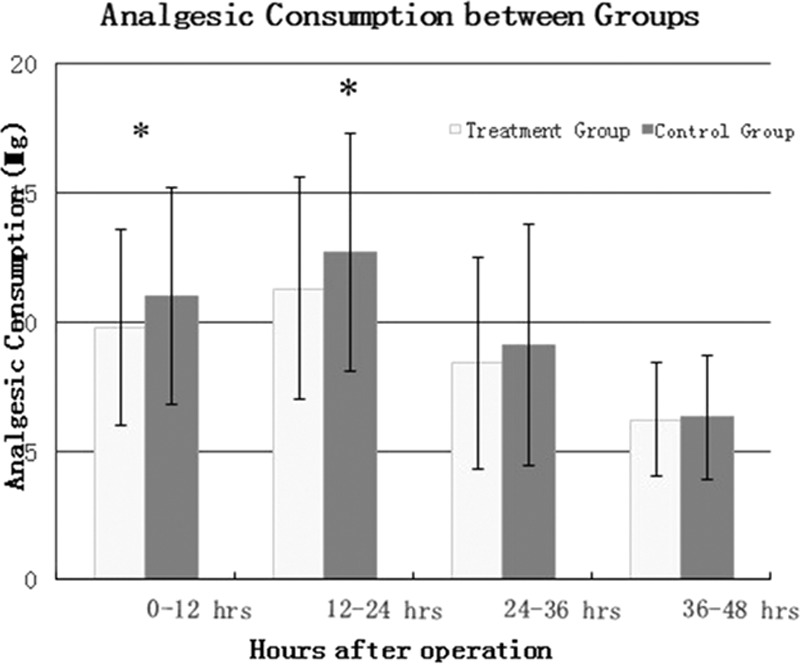
Analgesic consumption between two groups.

VAS pain scores in the treatment group showed a significant reduction at 4 hours (*P* = 0.0016), 8 hours (*P* = 0.0001), 12 hours (*P* = 0.0003), 16 hours (*P* = 0.0001), 20 hours (*P* = 0.0002), and 24 hours (P = 0.0004) (Figure 2). There was a significant reduction in analgesic consumption at stage 1 (0–12 hours, *P* = 0.0033) and 2 (12–24 hours, *P* = 0.0021) (Figure 3).

### Drainage Output, Decreased Hemoglobin, and Swelling

**FIGURE 4:**
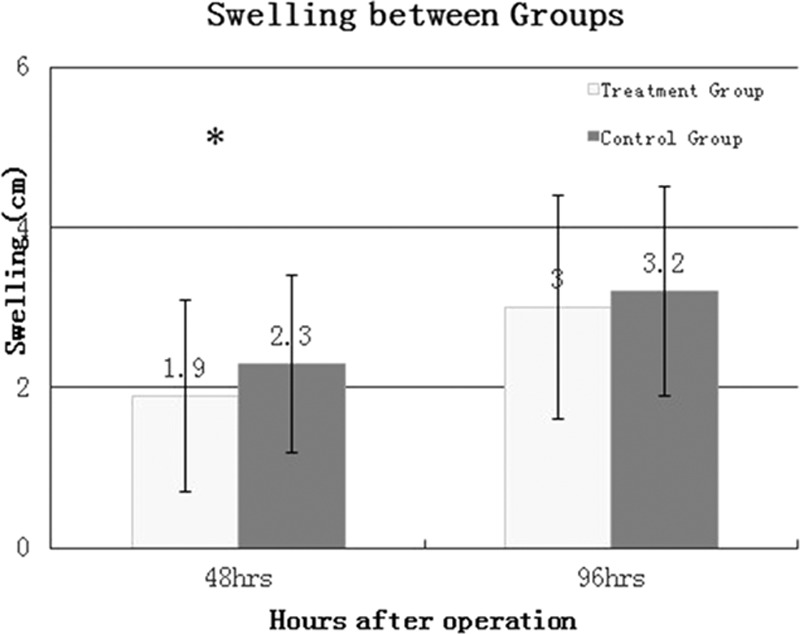
Swelling between two groups.

The drainage output in the treatment group was significantly less than the control group. Decreased hemoglobin in the control group was significantly lower compared with the treatment group (Table 2). A significant difference in knee swelling was found between groups at 48 hours after the procedure (*P* = 0.0007) (Figure 4).

**TABLE 2:**
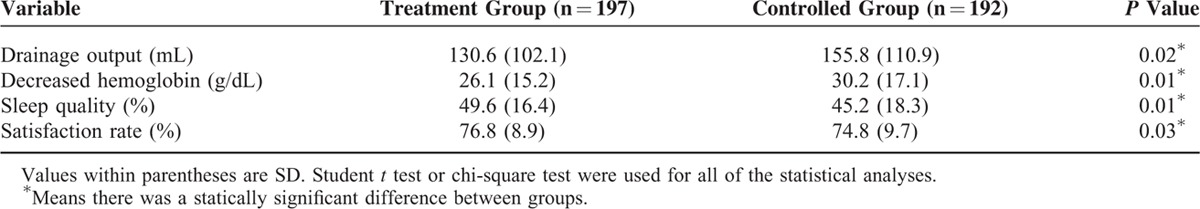
Clinical Outcomes of Participants after Operation

### Sleep Quality and Satisfaction Rate

Sleep quality and satisfaction rate were significantly higher in the treatment group than in the control group (Table 2).

### Complications and Side-Effects

No adverse events related to our medical intervention, including wound or deep infections, deep venous thrombosis (DVT), were observed throughout the study.

## DISCUSSION

In this randomized, double-blind, placebo-controlled and single-center study, we demonstrated that in patients undergoing TKA, an intraoperative application of 4000 mL chilled irrigation solution containing 0.5% epinephrine could improve the overall postoperative recovery. In our opinion, this safe, simple, and cost-effective intervention offers additional pain relief to the standard multimodal analgesic regime and a reduction in blood loss.

Previous studies have shown that pain peaked at 24 hours following TKA.^2,14^ As a result, sleep quality is severely impaired. Postoperative pain usually manifests at the initiation of the inflammatory response.^6^ Cryotherapy is believed to inhibit the inflammation mechanism caused by an increase in inflammatory cytokines and mediators.^13^ Traditionally, cryotherapy is applied postoperatively after an elevation in mediators and cytokines have initiated the inflammatory cascade.^3,15^ Consequently, the use of cryotherapy at the beginning of inflammatory reaction could reduce cytokines and mediators that induce inflammation. Few studies have investigated the effect of irrigation solution temperature in orthopedic procedures. In one study, cold irrigation did not significantly reduce postoperative pain and swelling in arthroscopic procedure.^2^ These findings might be because many arthroscopic procedures do not include administration of synovium, thus minimizing the effect on intra-articular inflammation.^2^ In addition, cold saline was withdrawn from the intra-articular tissues before the onset of the inflammatory response, as most of the irrigation was completed in <30 minutes. The characteristics of arthroscopic procedures are distinct from those of TKA procedures. The synovium, infrapatellar fat pad, and bursa, which are potential sources of pain, are partly or completely resected in TKA. Irrigation is applied after the completed osteotomy, for generally 30 to 40 minutes, and before the inflammatory response has begun. We designed irrigation with a capacity of 4000 mL consisting of 0.5% epinephrine cold and saline. About 50 mL of this was infused into the knee joint cavity after closure of the joint capsule. The use of 4000 mL cold saline is recommended because of its ability for removing particles during cemented TKA.

Local application of cold penetrates to a depth of 4 cm below the skin and induces clinical cyrotherapy.^16^ However, it is difficult for postoperative cryotherapy to freeze deep tissues in patients with a high body mass index (BMI) and swollen joints—a prevalent finding among TKA patients. In contrast, intraoperative cold irrigation is not affected by BMI. Cold irrigation solution is able to directly reach tissues in the articular cavity. The anesthetic effect of local cooling is produced by the slowing or elimination of pain signal transmissions.^3,7,15^ Our results revealed that VAS pain scores significantly decreased from 4 to 24 hours postoperatively with less analgesic use required. Swelling at 48 and 96 hours was not improved since it is difficult to maintain an effect for such an extended period from cold solutions. Therefore, continuous cryotherapy from intraoperative to postoperative points may provide a more efficacious option.

Cold stimulation is a safe vasoconstrictor and thus achieves a reduction in blood flow, which decreases the local inflammatory response and formation of edema.^3^ In addition, epinephrine also serves as a vasoconstrictor, and thus blood loss in our treatment group was significantly decreased.

Despite the limited benefits of pain relief and swelling in our study, the advantage of reduction in blood loss may be more significant and decrease the use and risks of blood transfusions. Symptoms of fatigue and pyrexia because of blood loss, which cam affect sleep quality and functional exercise, were reduced. In this subset of patients, sleep quality and satisfaction rates on the first postoperative night were significantly improved; and as a result, QOL during hospitalization was improved. In light of these possible advantages, our results revealed that irrigation is a safe, simple, effective, and economic treatment that can help realize the maximum benefits of TKA.

The prospective, randomized, double-blind, and controlled nature of this study, along with its large sample size, helps provide weight to our findings. This is the first study to focus on the effects of continuous irrigation on TKA results. Several major clinical outcomes of TKA were evaluated. In addition, we devised a 50 mL 0.5% epinephrine cold irrigation solution that was infused into the knee joint cavity by a drainage tube at the completion of suturing the joint capsule tissue to produce a more lasting effect.

However, there were several limitations to this study. It is not clear if these findings would remain true if cold irrigation was applied without a tourniquet in place. Additionally, the temperatures of the soft tissues after cold irrigation were not measured. The absence of these statistics may make the results relatively inaccurate. The degree to which the epinephrine solution affected intraoperative blood loss was not quantitatively measured. Furthermore, there seems to be a necessity to investigate whether intraoperative cold irrigation combined with cryotherapy after operation could obtain favorable benefits and be considered by future studies.

## CONCLUSION

Irrigation with a cold saline solution containing 0.5% epinephrine may benefit patients after TKA at a low cost. Such an application could significantly decrease postoperative VAS pain scores with reduced morphine consumption, improved sleep quality, and increased patient satisfaction after surgery. Additionally, reduction in blood loss may help decrease blood transfusion rates and their related costs. Collectively, this safe, simple, and effective technique may help recovery and enhance quality of life in patients undergoing TKA.
