# Effects of Cold Irrigation on Early Results after Total Knee Arthroplasty: A Randomized, Double-Blind, Controlled Study: Erratum

**DOI:** 10.1097/01.md.0000494762.95497.f6

**Published:** 2016-08-26

In the article “Effects of Cold Irrigation on Early Results after Total Knee Arthroplasty: A Randomized, Double-Blind, Controlled Study”,^1^ which appeared in Volume 95, Issue 24 of *Medicine*, Dr. Yong Wang's affiliation was originally listed incorrectly. The article has since been corrected online.
